# Quantitative analysis of superb microvascular imaging for monitoring tumor response to chemoradiotherapy in locally advanced cervical cancer

**DOI:** 10.3389/fonc.2022.1074173

**Published:** 2023-01-04

**Authors:** Yi Zhu, Yixin Tang, Guonan Zhang, Jie Zhang, Yanjie Li, Zhuolin Jiang

**Affiliations:** ^1^ Outpatient Department (Ultrasound), The Affiliated Cancer Hospital, University of Electronic Science and Technology of China, Sichuan Cancer Hospital and Institute, Chengdu, China; ^2^ Department of Ultrasound, Suining Central Hospital, Suining, China; ^3^ Department Gynecological Oncology, The Affiliated Cancer Hospital, University of Electronic Science and Technology of China, Sichuan Cancer Hospital and Institute, Chengdu, China; ^4^ Graduate School, Chengdu Medical College, Chengdu, China

**Keywords:** cervical cancer, chemoradiotherapy, ultrasound, superb microvascular imaging, vascularity index

## Abstract

**Objectives:**

As an ultrasound (US) image processing method, superb microvascular imaging (SMI) extracts and visualizes flow signals from vessels through advanced clutter suppression technology. We investigated the feasibility of SMI in monitoring treatment response in patients with locally advanced cervical cancer (LACC) undergoing chemoradiotherapy (CRT).

**Methods:**

Forty-nine patients underwent CRT and received SMI examination at 3 time points: before therapy (baseline), 3 weeks during, and 1 month after CRT. The maximum tumor diameter (Dmax), vascularity index (VI), and their percentage changes (ΔDmax and ΔVI) were calculated. ΔDmax was compared with MRI results as the reference standard.

**Results:**

Based on the MRI findings, 44 were classified as complete response (CR) group and 5 as partial response (PR) group. The Dmax and ΔDmax showed decrease in CR and PR groups at 3 weeks during CRT (P< 0.05), but no significant difference between the two groups (P > 0.05). Compared to the baseline, significant decrease in VI and ΔVI were observed at during and after treatment in the two groups (P< 0.05). Moreover, there were significant differences in VI and ΔVI at 3 weeks during CRT between the CR and PR groups (P< 0.05). ΔVI at 3 weeks during CRT showed a better predictive performance for responder prognosis than VI (AUC = 0.964, AUC = 0.950, respectively, P = 0.001), with a cut-off value of 41.6% yielding 100% sensitivity and 86.4% specificity.

**Conclusions:**

The SMI parameters (VI and ΔVI) have potential for monitoring treatment response in LACC.

## Introduction

Cervical cancer, as the fourth most common malignancy, has become a global female health problem. Cervical cancer is estimated to cause 570,000 new cases and 311,000 deaths each year (1, 2). At the same time, the onset age of cervical cancer tends to be younger, from the original 40-50 years old to 35 years old, with an annual increase of 2%-3%. Especially in low- to middle-income countries (LMIC), where lack the screen and adequate treatment, approximately 90% of cervical cancer remains fatal (3). Locally advanced cervical cancer (LACC) (FIGO stage IB2-IVA) has the characteristics of large lesions (> 4 cm), easy distant metastasis, difficult to operate directly, and poor therapeutic effect. Concomitant chemotherapy and radiotherapy (CRT) consisting of cisplatin-based chemotherapy, external-beam radiotherapy (EBRT), and intracavitary brachytherapy (ICR) is considered the recommended standard treatment for LACC (4). Due to tumor heterogeneity, all cancers are unlikely to respond uniformly to a specific treatment regimen, resulting in tumor uncontrolled, locoregional recurrence, or distant metastases after treatment in some patients (5, 6). Thus, surveillance of changes in tumor burden associated with treatment will be helpful for adjusting treatment strategy to obtain a better outcome in LACC (7, 8).

Current conventional imaging techniques, such as magnetic resonance imaging (MRI), computed tomography (CT) and ultrasound (US), rely on identifying morphological changes to evaluate and monitor the effect of CRT or disease progression in LACC. In fact, changes at the molecular or cellular level that occur early in responders significantly precede changes in tumor volume or size (9). Microstructural and microcirculatory changes during anticancer therapy can be detected by functional imaging (18F-fluorodeoxyglucose positron emission tomography, dynamic contrast-enhanced MRI, diffusion-weighted MRI, et al.) (10–12). However, these new approaches have limitations such as increased radiation burden, potential reaction effects of contrast agents, high cost, or technical complexity, making them difficult to be used for monitoring in the clinical routine (9, 13). Therefore, based on efficacy, safety and health economics considerations, US remains the preferred method for tracking curative effect, especially in LMIC.

Increased vascularization plays a crucial for sustain tumor growth, invasion, and metastasis (14, 15). It is demonstrated that angiogenesis is an important factor affecting cervical cancer development and survival prognosis (16, 17). Some studies have reported that the degree of tumor vascularity decline is directly proportional to therapeutic response. Thus, the assessment of tumor vascularity could become a novel means of monitoring tumor response to CRT in LACC.

Color or power Doppler US plays an indispensable role in assessing tumor angiogenesis and predicting the efficacy of CRT in cervical cancer (18, 19). Unfortunately, color or power Doppler US is limited by a wall filter to truly distinguish between low-flow components and clutter motion artifacts, which makes the fine vessels of cervical lesions potentially undetectable. In addition, color or power Doppler US has demonstrated poor reproducibility (20). With the advent of intravenous US contrast agent, contrast-enhanced US (CEUS) significantly enhances the signal of slow and low-volume blood flow to improve the visualization of with microvascular (20 - 39 μm in diameter) (21). Cervical cancer has markedly different quantitative and qualitative filling patterns with CEUS. CEUS might be a valuable tool in predicting remaining tumor on treatment (22). However, CEUS is an invasive imaging and carries the risk of drug allergy. Superb microvascular imaging (SMI) is a unique ultrasonic Doppler technology. SMI extracts and visualizes flow signals from vessels through advanced clutter suppression technology, enabling clear visualization of low velocity small-volume blood vessels without the use of contrast agents. Under the qualitative guidance of SMI images, tumor angiogenesis was quantitatively evaluated by vascular index (VI) (23). Although some preliminary experience reports have demonstrated the benefits of SMI in the diagnosis of multiple tumors (23, 24), the potential of this new US technique for efficacy assessment in cervical cancer has not yet been fully evaluated.

We aimed to assess whether using SMI to evaluate tumor vascularity could provide a means for monitoring tumor response to CRT in a series of patients with LACC.

## Materials and methods

### Patients and treatment

**Figure 1:**
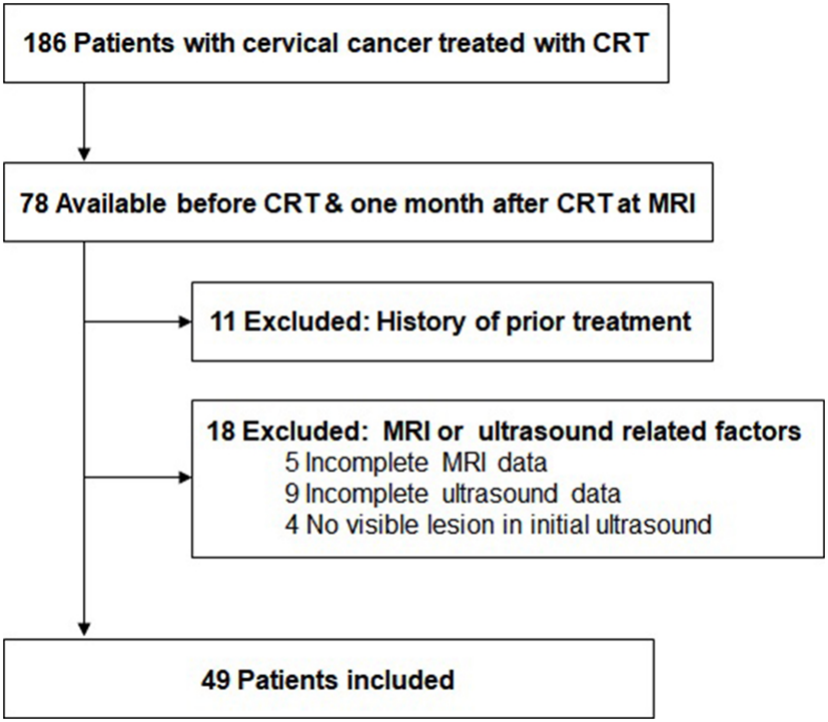
Flowchart of patient enrollment. CRT, chemoradiotherapy.

Forty-nine patients with LACC (FIGO stage IIB to IVA) were enrolled from September 2020 and May 2022. The exclusion criteria were follows: (a) history of prior treatment; (b) dropping out during therapy; (c) incomplete US or MRI data (**Figure 1**). All patients received histopathological diagnosis through multiple punch biopsies and had complete medical records.

All patients underwent external beam radiotherapy (EBRT) with 15-MV photon beams at a daily dose of 1.8-2.0 Gy (5/w), with a median total dose of 50.4 Gy. Subsequently, high-dose-rate intracavitary brachytherapy (ICR) was performed twice a week with an iridium-192 source as 6 Gy per insertion in five fractions (a total dose of 30 Gy). Four to six cycles of platinum-based chemotherapy were supplemented at 3-week intervals. The formulation of chemotherapy cycle is generally determined by various factors such as FIGO stage, malignancy, and physical condition.

### Treatment response evaluation

MRI is the best currently acceptable and reproducible method to assess objective response in cervical cancer. Whole-pelvic cavity and perineum MR data from patients with LACC were obtained from 3.0T MR scanner (Siemens Magnetom Skyra, Germany) within one week before treatment and 1 month after therapy completion. The longest tumor diameter was analyzed according to sagittal T2-weighted images and the percentage change in tumor size was calculated as follows:


$$Change\text{ in }tumor\;size\%=\frac{\left(pre-longest\;diameter-post-longer\;diameter\right)}{pre-longest\;diameter}\times 100\%$$

The clinical efficacy was assessed by two radiologists with more than 5 years of experience in pelvic MRI diagnosis according to the Response Evaluation Criteria for Solid Tumors (RECIST) guidelines (version 1.1) (25). The radiologists reached consensus through discussion to resolve differences in image interpretation. All patients were divided into four groups: complete response (CR) as disappearance of all target lesions; partial response (PR) as a reduction in tumor diameter of more than 30%; progressive disease (PD) as an increase in tumor diameter of more than 20%; stable disease (SD) as neither sufficient regression to match PR nor sufficient enlargement to match PD (25).

### Transvaginal ultrasound examination

**Figure 2:**
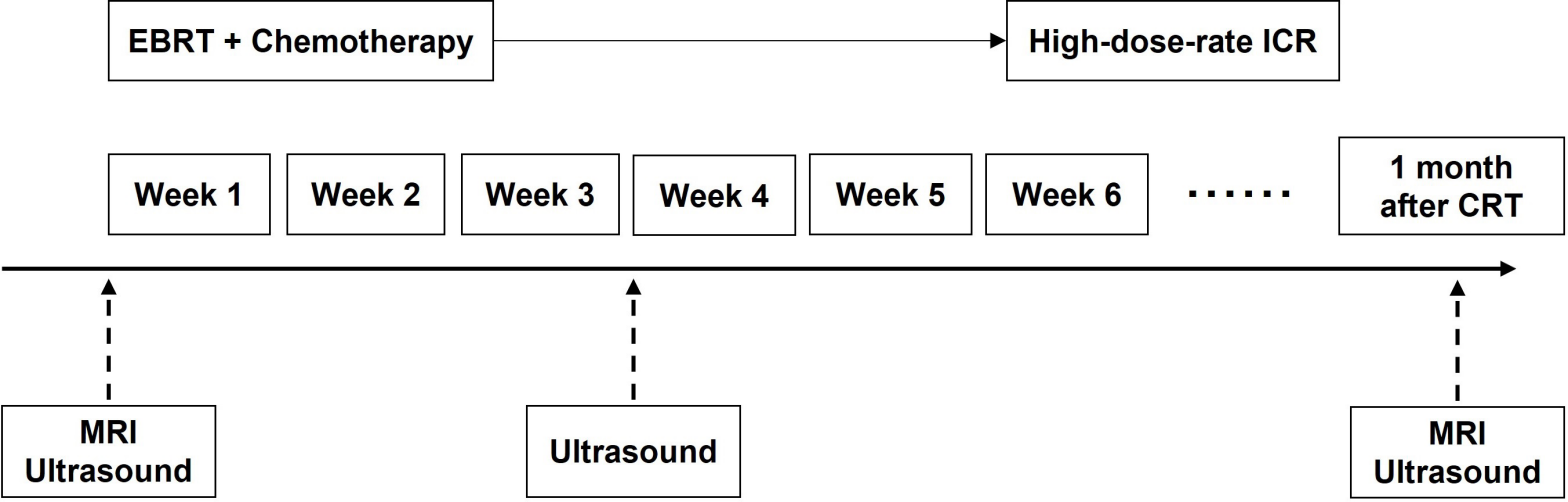
The schedule of MRI, TVUS examination and treatment. CRT, chemoradiotherapy; EBRT, external beam radiotherapy; ICR, intracavitary brachytherapy.


All real-time transvaginal US (TVUS) examinations and the SMI analysis were performed by a group of three fellows with more than 8 years of experience in US of gynecological oncology, who were unaware of MRI findings and the treatment outcome. TVUS examination was performed at 3 time points: pre-therapy (baseline), 3 weeks during (mean, 18.4 days; range, 15 - 21 days), and 1 month after CRT (**Figure 2**), using an Aplio i800 US system (Canon Medical Systems, Tokyo, Japan) with a multifrequency linear 3 - 11 MHz endovaginal transducer. All SMI examination were acquired with the same settings throughout the study: 8.5 cm depth, 3.5 focal zone, 5.8 MHz Doppler frequency, 43 color gain, frame rate > 50 fps, to ensure quantitative US comparison. VI value was obtained by manually delineating the lesion (or cervix) boundary in a still SMI image with the maximum Doppler signals. One fellow performed real-time US including TVUS and measurement of the VI of SMI, followed by the other two fellows obtaining additional VI measurements for the lesion, then taking the average value. The change in VI was calculated based on the following formula:

$$Change\;in\;VI(\Delta VI)=\frac{\left(VIpre-VIpost\right)}{VIpre}\times 100\%$$

The change in the maximum long-axis diameter (Dmax) of the primary tumor was calculated to quantitatively evaluate the efficacy of CRT, according to the following formula:


$$Change\;in\;VI(\Delta Dmax)=\frac{\left(Dmaxpre-Dmaxpost\right)}{D\text{maxpre}}\times 100\%$$

### Statistical analysis

Statistical analysis was put into action on SPSS software version 22.0 (SPSS Inc., Chicago, IL, USA). The difference in characteristics of the two groups was compared using Student’s unpaired t-test or Mann-Whitney test. The multiple comparisons in VI, tumor Dmax and their changes between the two groups were assessed using repeated measures analysis of variance (ANOVA) and Student’s unpaired t-test. Receiver operating characteristic (ROC) curve and area under curve (AUC) were applied to analyze the value of each index in predicting CRT. Intraclass correlation coefficients (ICCs) were calculated to estimate interobserver and intraobserver reproducibility. The ICC value was judged to provide excellent reliability (0.81 - 1.00), good reliability (0.61 - 0.80), moderate reliability (0.41 - 0.60), or poor reliability (0.00 - 0.20), fair reliability (0.21 - 0.40). Two-sided test was used for all tests, and p< 0.05 was considered statistically significant.

## Results

### Patient and tumor characteristics

**Figure 3:**
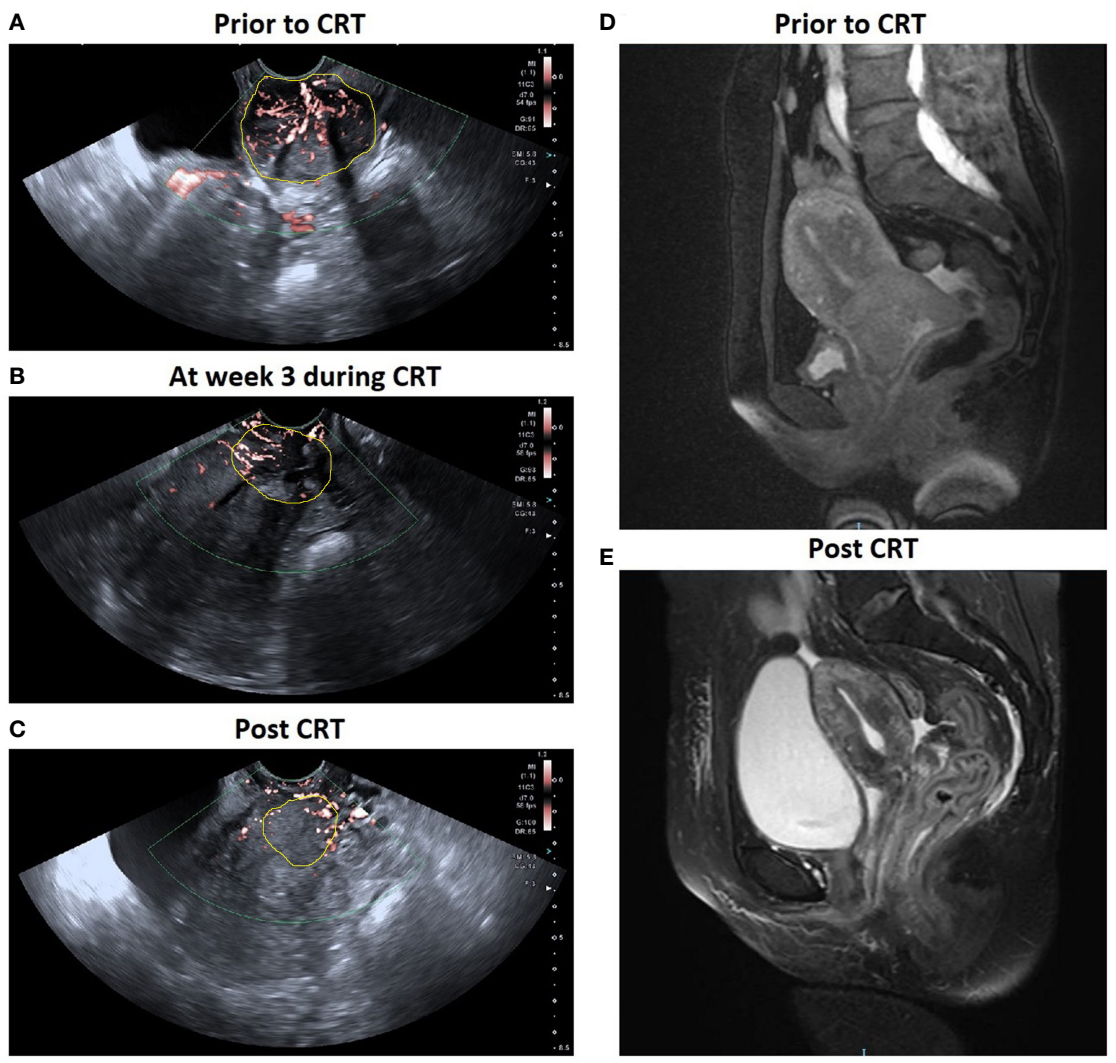
A patient with locally advanced cervical cancer (FIGO stage IIIB) experienced complete response to chemo-radiotherapy (CRT). SMI images show a significant decrease in VI in cervical cancer: **(A)** 0.367 prior to CRT; **(B)** 0.188 at week 3 during CRT; **(C)** 0.600 post CRT. Corresponding axial T2-weighted images exhibited a significant decrease in the maximal diameter of tumor: **(D)** 5.3 cm at pre-therapy e and **(E)** 0 cm post therapy.

**Figure 4:**
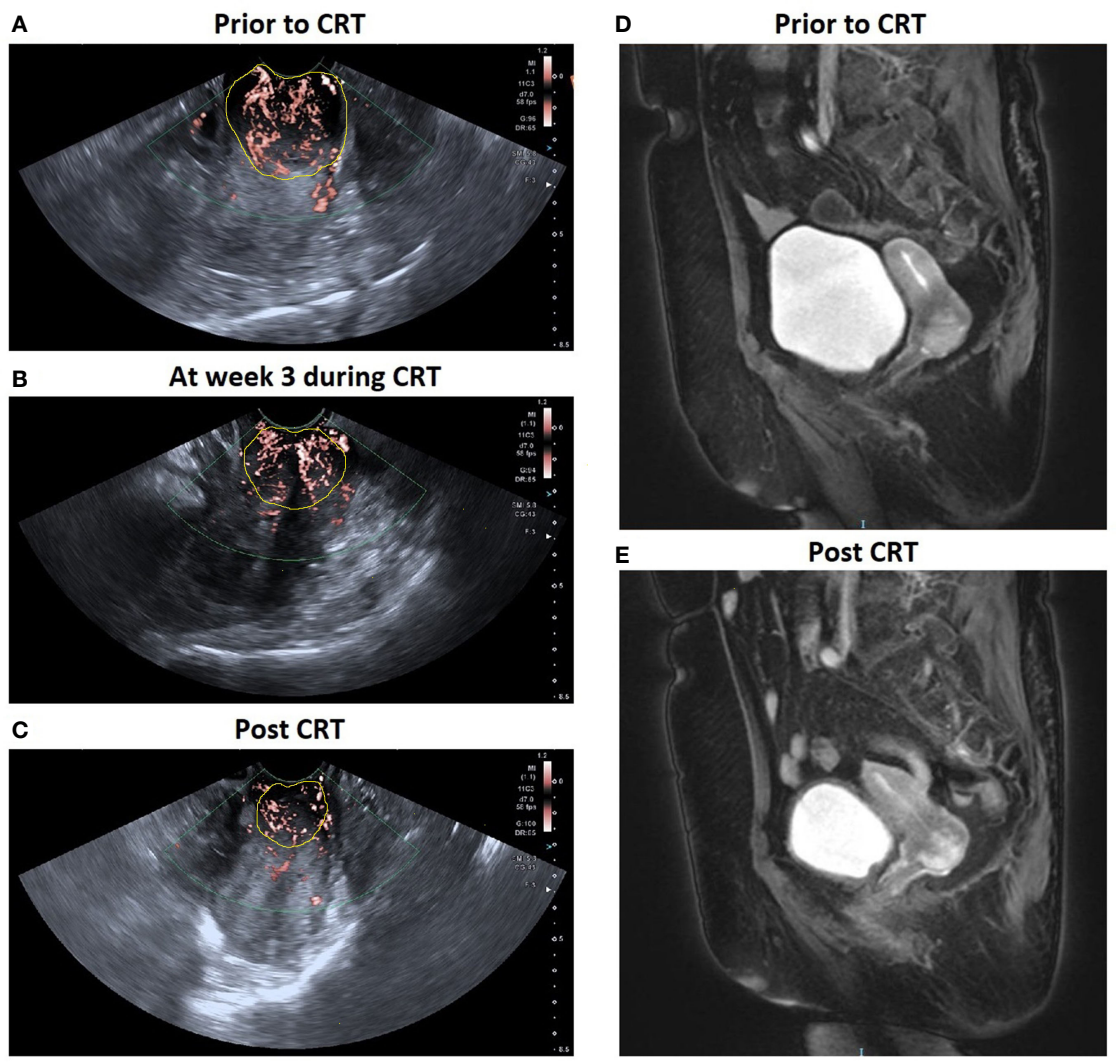
A patient with locally advanced cervical cancer (FIGO stage IIB) experienced complete response to CRT. SMI images show a significant decrease in VI in cervical cancer: **(A)** 0.398 prior to CRT; **(B)** 0.323 at week 3 during CRT; **(C)** 0.278 post CRT. Corresponding axial T2-weighted images exhibited a significant decrease in the maximal diameter of tumor: **(D)** 3.5 cm at pre-therapy e and **(E)** 1.8 cm post therapy.

The characteristics of the study patients are summarized in **Table 1**. The mean age of the 49 patients was 54 ± 10.7 years, ranging from 33 to 72 years. Based on the MRI findings, 44 were classified as CR group, 5 as PR group, and 0 in SD/PD groups. The mean age was 54.1 ± 11.0 years for the CR group and 52.8 ± 9.3 years for the PR group (p = 0.636). There was no significant difference between the CR and PR groups in the primary tumor Dmax (p = 0.147), FIGO stage (p = 0.504), histological type (p = 0.554) or histological grade (p = 0.636). **Figure 3** illustrated the SMI images and corresponding axial T2-weighted MRI of a typical CR case throughout the treatment, while showed **Figure 4** a representative PR case.

**Table 1:** Characteristics of the study group.

Characteristics	No. of patients (%)
Age (years)	
** ≤ 50**	14 (28.6)
** 51 - 60**	20 (40.8)
** > 60**	15 (30.6)
FIGO stage	
** IIB**	10 (20.4)
** IIIA**	15 (30.6)
** IIIB**	12 (24.5)
** IIIC**	8 (16.3)
** IVA**	4 (8.2)
Histologic type	
** Squamous cell carcinoma**	42 (85.7)
** Adenocarcinoma**	7 (14.3)
Histological grade	
** G1 - G2**	19 (38.8)
** G3**	30 (61.2)
Tumor diameter (primary)	
** < 4 cm**	8 (16.3)
** ≥ 4 cm**	41 (83.7)
Treatment outcome	
** Complete response**	44 (89.8)
** Partial response**	5 (10.2)

G1, well differentiated; G2, moderately differentiated; G3, poorly differentiated; FIGO, the International Federation of Gynecology and Obstetrics.

### Predictive values of tumor size and changes during CRT

At 3 weeks during CRT, Dmax of CR group was slightly lower than that of PR group (p = 0.083), and CR group had higher ΔDmax than PR group (p = 0.435), but there were no significant different between the two groups. After treatment, Dmax was significantly reduced in both CR and PR groups. Decrease in Dmax of CR group (ΔDmax = 100%) is obviously more than that of PR group (ΔDmax = 47.3 ± 6.8%) (p< 0.001) (**Table 2**). The results of ΔDmax of the two groups showed good agreement with MRI. The ROC analysis revealed no significant area-under-the-curve (AUC) values for Dmax (AUC = 0.745, p = 0.087) and ΔDmax (AUC = 0.536, p = 0.792) at 3 weeks during CRT (**Figure S1**).

**Table 2:** The mean Dmax and VI value of the tumor in the complete and partial responders at each time point.

Time-points	Dmax (cm)		*P*	VI		*P*	ΔDmax (%)		*P*	ΔVI (%)		*P*
	CR	PR		CR	PR		CR	PR		CR	PR	
**Pre-therapy (Baseline)**	5.1 ± 1.1	5.9 ± 1.4	0.147	0.400 ± 0.060	0.401 ± 0.032	0.970						
**3 weeks during CRT**	3.1 ± 0.9	3.8 ± 0.7	0.083	0.237 ± 0.045	0.320 ± 0.014	0.004	38.3 ± 14.6	32.8 ± 17.4	0.435	40.8 ± 6.7	19.6 ± 9.3	<0.001
**1 month after CRT**	0	3.1 ± 1.0	<0.001	0.072 ± 0.019	0.291 ± 0.023	<0.001	100	47.3 ± 6.8	<0.001	82.2 ± 3.5	26.6 ± 10.9	<0.001

Data are presented as mean ± standard deviation.

CR, complete responder; PR, partial responder; CRT, Concomitant chemotherapy and radiotherapy; Dmax, the maximum long-axis diameter; VI, vascularization index.

### Predictive values of VI and changes during CRT

**Table 2** summarizes the mean VI and ΔVI of the tumors in the CR and PR groups at each time point. Before starting treatment, CR group and PR group demonstrated similar tumor angiogenesis, with mean VI of 0.400 ± 0.060 and 0.401 ± 0.032, respectively. VI decreased during CRT (p< 0.001) in patients with LACC. The VIs were significantly decreased from 3 weeks after treatment initiation to therapy completion in the CR group (p< 0.001), and the difference between CR and PR groups was found to be significant (p = 0.004 and p< 0.001). Compared to baseline at pre-therapy, VI of the PR group slightly decreased at 3 weeks during treatment (p = 0.007). However, no significant difference of VI was seen from 3 week after treatment initiation to therapy completion in PR group (p = 0.078). Similarly, ΔVI exhibited significant differences between the two groups at 3 weeks during treatment and after treatment (all p< 0.001). The VI as well as ΔVI at 3 weeks during CRT was able to predict the responder prognosis, with an AUC of 0.950 (p = 0.001) and 0.964 (p = 0.001), respectively. The optimal cut-off values for predicting responder prognosis were 0.264 for VI and 41.6% for ΔVI, respectively. Furthermore, ΔVI at 3 weeks during treatment showed a better predictive performance for responder prognosis than VI, with a 100% sensitivity and 86.4% specificity (**Figure S1**).

### Reproducibility analysis

The ICCs of VI for interobserver and intraobserver variability of measurements were 0.911 (95% CI, 0.885 - 0.932; p< 0.001) and 0.925 (95% CI, 0.910 - 0.937; p = 0.001), respectively.

## Discussion

CRT improves long-term survival and reduce local recurrence in patients, which is one of the most important methods of treatment for LACC (26). Due to cytotoxic effects, chemotherapeutic drugs (such as cisplatin and paclitaxel) act directly on microvascular and tumor cells to reduce tumor size, leading to tumor re-oxidation and cell cycle entry into a radiation-sensitive phase. Currently, imaging modalities are used to effectively evaluate tumor size change to intuitively reflect the treatment effect. We observed significant decreases in tumor size during and after CRT in patients with LACC, especially in the CR group. Although US is not recommended as a general tool in RECIST guideline (27), our study confirmed that tumor size reduction on US images were largely consistent with that on MRI images after CTR. However, there was no significant difference in tumor size between the CR and PR groups at 3 weeks during treatment, which did not appear to be useful in predicting tumor response at initial stage of treatment. However, reduction in tumor size may occur after several weeks, despite a positive functional response to therapy (28). Thus, an early and accurate predicting marker of effective therapy is needed to provide a basis for clinical optimization of treatment regimens, while also reducing unnecessary post-treatment toxicities and economic costs.

Highly vascularized tumors are more aggressive and have a poorer prognosis than less vascularized tumors, indicating that highly vascularized tumors may be more resistant to chemotherapy and radiotherapy. With the gradual regression of vascularization during CRT, the tumor structure changed and size decreased step in step (29). Changes of vascularization are potential non-invasive markers for tumor response forecast to CRT in patients with cervical cancer (30). According to previous studies, using transvaginal color Doppler US (TVCD), 3-dimensional power Doppler angiography (3D-PDA) and CEUS to evaluate tumor vascularity correlates with some tumor features and could provide a means for predicting clinical response to CRT in patients with LACC (18, 19, 28, 31). Moreover, it is demonstrated that strain elastography was useful as an early predictor of respond and long-term outcomes after CCRT for patients with cervical cancer (13). But, there was no consensus on the effectiveness of SMI and strain elastography in the evaluation of tumors and treatment response (32, 33).

SMI can detect micro-vessels with diameter as small as 0.1 mm or low-velocity blood flow (≥ 0.8 cm/s), and is therefore considered a promising, low-cost, and safe method for imaging angiogenic changes in tumors. In several studies, SMI has significant advantages over color and power Doppler imaging in detecting ultra-low velocity blood flow in microvessels and blood microperfusion of tumor (34, 35). For evaluation tumors by assessing the microvasculature, the diagnostic performance of SMI appears to be comparable to that of CEUS (24). SMI quantitative analysis showed VI was in direct proportion to the vascularization. We found that the VI showed a significant downward trend compared with that before treatment. SMI is expected to be a reliable method for monitoring intratumor microvascular density changes after CRT. Our results showed changes in quantitative SMI parameters (VI) indirectly reflected the effectiveness of CRT, which were consistent with previous reports using TVCD and 3D-PDA technology.

Multiple studies have shown that CEUS revealed changes in tumor blood flow pattern during treatment precede anatomical changes detected by imaging (28, 36). However, CEUS is difficult to implement as a routine technique to track efficacy in daily clinical practice. Our study investigated whether VI discriminated between CR and PR groups at baseline and following CRT. We found that the VIs of CR decreased more significantly than that of PR group at 3 weeks during treatment in the condition that there were no significant changes in tumor size between the two groups. Our findings indicated that the significant size reduction usually occurs at a later stage of treatment, so the changes of tumor microvasculature can be detected by SMI quantitative analysis to reflect the early therapeutic effect.

The heterogeneity of tumor microvasculature distribution determines the efficacy of anticancer drugs in killing tumor cells by affecting the delivery of therapeutic drugs. Hypervascular tumors allow enough anticancer drugs to penetrate deeper into the tumor and be destroyed, whereas hypovascular tumors may be more likely to survive due to exposure to lower drug concentrations (37). Our study demonstrated that baseline VIs was slightly higher in the CR group than in the PR group before starting treatment, but no significant differences were obtained. Due to the varying sample size of each group, the statistical results may be biased. Although SMI quantitative analysis can reflect the blood supply of tumor tissue, we cannot conclude that the tumor response to CRT can be predicted by baseline VI. Thus, further investigation will be needed to verify whether baseline VI can guide the selection of pretreatment strategies for cervical cancer and be is a predictor of antiangiogenic therapy.

Our research has some limitations. First, this study was a single-center retrospective study with a small sample size, which may have limitations such as sample selection bias, short observation time. Second, due to the lack of long-term follow-up results of SMI in predicting clinical outcomes in LACC patients, further studies are needed. Finally, signal intensity of SMI is strongly dependent on depth and patient weight. We used the same US unit and setup for each patient, and as far as possible ensured that the tumor changes were evaluated with similar anatomical sections throughout the treatment, so the measurements at 3 time points were comparable.

## Conclusion

SMI quantitative analysis can reflect changes in tumor microvasculature, which likely precede changes in tumor size following CRT. SMI is emerging as promising valuable vascular imaging technique for monitoring tumor response to CRT in LACC. Of course, multicenter, large-sample, controlled studies are needed to further investigate the role of SMI in monitoring clinic outcome in patients with LACC underwent CRT.

## Data availability statement

The original contributions presented in the study are included in the article/**Supplementary Material**. Further inquiries can be directed to the corresponding author. A small sample of the study subjects has been used in previous reports at the 2021 RSNA (Yi Zhu. Superb Microvascular Imaging: Preliminary Results in Assessment the response to Chemoradiotherapy in Locally Advanced Cervical Cancer. 2020 RSNA. Abstract ID: 2021-SP-18091-RSNA). In this paper, we added more cases and enriched the analysis.

## Ethics statement

Written informed consent was not obtained from the individual(s) for the publication of any potentially identifiable images or data included in this article.

## Author contributions

YZ and GZ conceived, designed, or planned the study; YZ, YT, and JZ provided study patients, imaging and pathological data; YT and YL collected or assembled the data; YZ and YT performed or supervised analyses; YZ wrote sections of the initial draft; YZ and GZ provided substantive suggestions for revision or critically reviewed subsequent iterations of the manuscript; GZ provided administrative, technical, or logistic support. YZ and YT contributed equally to this work. All authors reviewed and approved final version of the paper; are accountable for all aspects of the work in ensuring that questions related to the accuracy or integrity of any part of the work are appropriately investigated and resolved.

## Supplementary material

Supplementary Figure 1 Receiver-operating characteristic (ROC) curve of morphological changes during the CRT. Maximum tumor diameter (Dmax, **A**) and their percentage changes (ΔDmax, **B**) cannot predict the long-term prognosis at 3 weeks during CRT. Vascularity index (VI, **C**) and ΔVI **(D)** can predict the long-term prognosis at 3 weeks during CRT.
