# Supplementary figures and images for: Quantitative analysis of superb microvascular imaging for monitoring tumor response to chemoradiotherapy in locally advanced cervical cancer
Source: Front Oncol. 2023 Jan 4;12:1074173. doi: 10.3389/fonc.2022.1074173 (PMC9848652; doi:10.3389/fonc.2022.1074173)

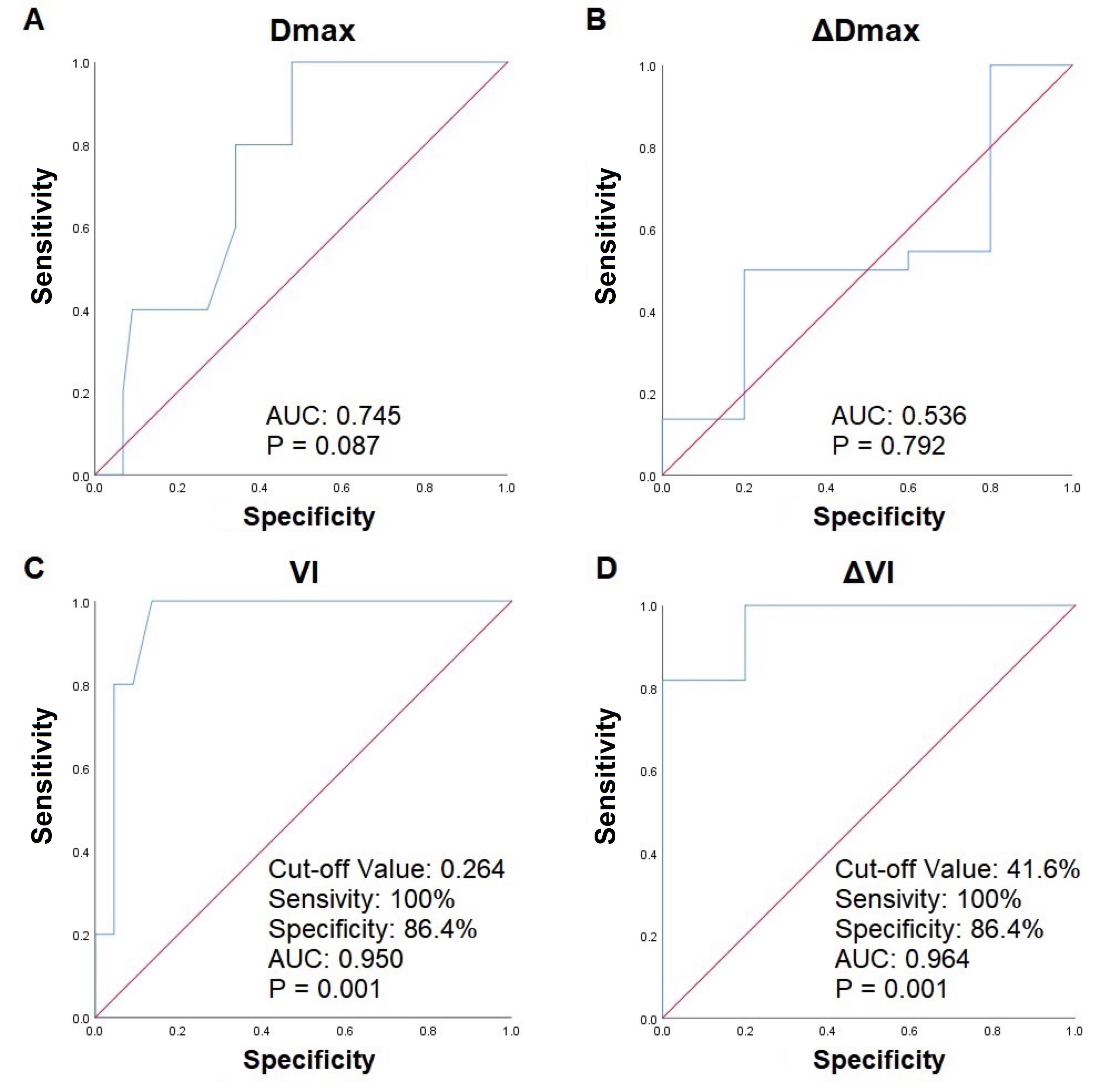

Supplement: Supplementary Figure 1 — Receiver-operating characteristic (ROC) curve of morphological changes during the CRT. Maximum tumor diameter (Dmax, A) and their percentage changes (ΔDmax, B) cannot predict the long-term prognosis at 3 weeks during CRT. Vascularity index (VI, C) and ΔVI (D) can predict the long-term prognosis at 3 weeks during CRT. [file Image_1.jpeg]
